# Plasma Proteome Profiling Identifies Biomarkers and Potential Drug Targets for Non-Small Cell Lung Cancer

**DOI:** 10.7150/ijms.107109

**Published:** 2025-09-12

**Authors:** Minghui Zhao, Xiaoke Di, Yucui Zhao

**Affiliations:** 1 Department of Radiation Oncology, The First Affiliated Hospital with Nanjing Medical University, Nanjing, China.; 2 Department of Radiation Oncology, The Second Affiliated Hospital, Zhejiang University School of Medicine, Hangzhou, China.

**Keywords:** proteome-wide Mendelian randomization, biomarker, drug target, non-small cell lung cancer.

## Abstract

Non-small cell lung cancer (NSCLC), as one of the most commonly diagnosed cancers globally, requires expedited identification of new drug targets. We conducted proteome-wide MR using genetic data for 4,853 plasma proteins. Summary-level data on lung adenocarcinoma (LUAD) and squamous cell carcinoma (LUSC) were extracted from GWAS meta-analyses (11,273 and 7,426 cases, respectively) and FinnGen cohort (1,590 and 1,510 cases, respectively). We genetically identified eight proteins with a causal role in the etiology of NSCLC. Lower levels of five proteins (CDH17, CXADR, FAM3D, POGLUT3, SFTPB) and higher levels of two proteins (CEACAM5, KLK1) were linked to increased LUAD risk, while higher CD14 levels were associated with elevated LUSC risk. Two proteins, POGLUT3 and SFTPB were validated through Bayesian colocalization. One protein SFTPB was identified using SMR and HEIDI tests. Bidirectional MR found no reverse causality. The primary findings were validated through scRNA-seq, GeneMANIA, GO analysis, druggability assessments and PheWAS analysis. These protein-coding genes are primarily expressed in epithelial cells, macrophages, monocytes, and endothelial cells. Furthermore, CEACAM5, KLK1, and CD14 correspond to existing drugs. These proteins may deepen our comprehension of the etiology and could serve as appealing novel biomarkers and drug targets for NSCLC management.

## Introduction

Lung cancer is the most frequently diagnosed malignancy globally and is the leading cause of cancer-related death. Annually, it is estimated that around 2.5 million new cases arise and over 1.8 million fatalities occur 1. Approximately 80% to 85% of lung cancers are non-small cell lung cancer (NSCLC), with lung adenocarcinoma (LUAD) and lung squamous cell carcinoma (LUSC) being the two predominant subtypes. LUAD has become the most common subtype, with incidence rates exceeding those of LUSC in recent years 2. Most patients are diagnosed at an advanced stage, where curative treatment options are limited, resulting in a 5-year survival rate that hovers just above 20% 3, 4. Therefore, there is an urgent need to discover sensitive and specific markers for the early prediction of lung cancer to improve outcomes. Additionally, the development of novel therapeutic strategies is essential, either as standalone interventions or to enhance current prevention programs such as smoking cessation 5.

Proteins serve as the cornerstone of human metabolism, translating genomic information into the processes of growth, development, and homeostasis. The levels and characteristics of individual plasma proteins reflect a range of physiological or pathological states. Their accessibility via minimally invasive and inexpensive methods renders them ideal candidates for biomarker identification and as targets for drug development 6. The advent of high-throughput technologies has propelled research into the potential of plasma proteins as predictors of lung cancer 7, 8. These proteins are particularly promising as drug targets, as their interaction with therapeutic agents can be efficiently monitored in the bloodstream during randomized controlled trials (RCTs), thereby accelerating the drug development pipelines. Despite these advances, there remains a significant gap in meeting the clinical demand for novel biomarkers.

Mendelian randomization (MR) is a technique that leverages genetic variants as natural instruments to establish putative causality between phenotypes, thereby minimizing the potential for residual confounding and reverse causation bias in observational studies 9. It relies on three key hypotheses: (i) the instrumental variable is associated with the exposure (relevance), (ii) the instrumental variable is not associated with any confounders (independence), (iii) the genetic instrument should only influence the outcome through the exposure, not through any other pathways, thus isolating the effect of the exposure on the outcome (exclusion restriction).

Human genetic variation has wide-ranging effects on protein structure and function, with important implications for health and disease. Proteomics data research through protein quantitative trait loci (pQTL) has opened new avenues for biomarker discovery and revealing disease mechanisms 10. Integrating proteomics with genomics and MR has facilitated the identification of the causal roles of proteins in diseases, providing insights into etiology and potential therapeutic targets for colorectal cancer, ovarian cancer, stroke, and psychiatric disorders 11-14.

**Figure 1:**
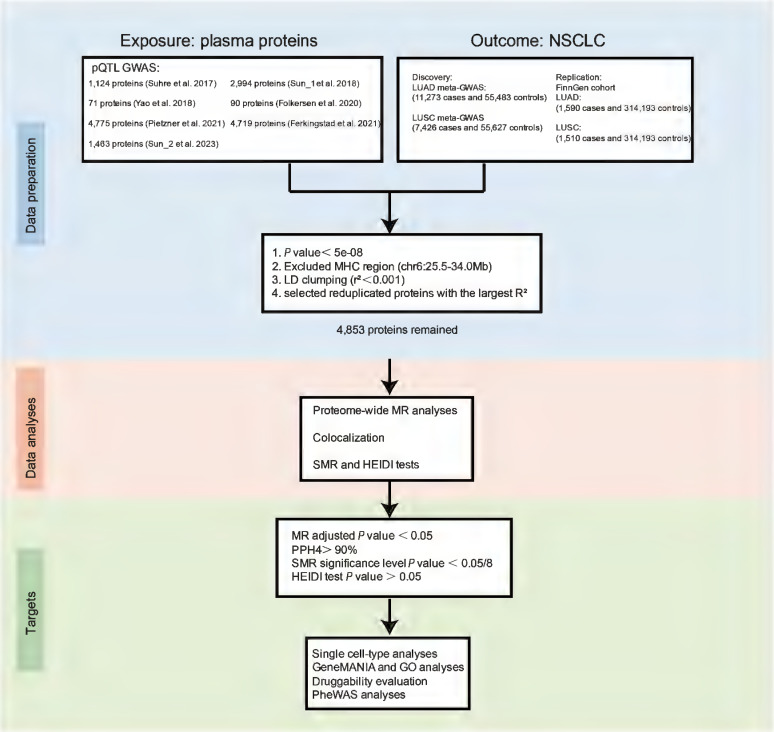
Overall flow chart of the study.

In the current study, we conducted proteome-wide MR analyses utilizing pQTL summary statistics from seven large-scaled proteomic studies. The study design is illustrated in Figure 1. This approach was designed to pinpoint circulating protein markers with a causal link to NSCLC risk. Bayesian colocalization, SMR (summary-data-based Mendelian randomization) and HEIDI (heterogeneity in dependent instruments) tests were also used to robustly identify cancer-risk proteins. Then single cell-type expression analysis was employed to detect the specific enrichment of these proteins in lung cancer tissue. Furthermore, we assessed the expression levels of these protein-coding genes between cancer and normal lung tissue using the GEPIA database. GeneMANIA and Gene Ontology (GO) analysis were conducted to explore the potential genetic interactions and functions of identified protein-coding genes. This was followed by a thorough druggability evaluation aimed at determining their potential as therapeutic targets for lung cancer. Lastly, risk proteins were mapped to potential therapeutic interventions and phenome-wide association study (PheWAS) was employed to elucidate the potential benefits and challenges of intervening on these proteins, including the possibility of unforeseen adverse outcomes. These multifaceted analyses not only provide compelling causal evidence for the role of proteins in lung cancer genesis, but also offer a new perspective on the predictive and preventive strategies for lung cancer.

## Materials and Methods

We performed proteome-wide MR based on pQTLs from 7 large-scale proteomic studies and summary genetic association data of NSCLC from previously published GWAS, to examine the causal proteins associated with LUAD and LUSC. All contributing studies included in this analysis were approved by the relevant institutional review board from each country and all participants provided informed consent.

### Study data sources and selection of instrumental variables (IVs)

The GWAS data for NSCLC in the primary outcome were obtained from a large-scale GWAS study of European descent, including 66,756 individuals of LUAD (11,273 cases and 55,483 controls) and 63,053 individuals of LUSC (7,426 cases and 55,627 controls), respectively 15. In addition, for external validation, we used the latest release data on NSCLC from the FinnGen study R10 in this analysis, comprising 1,590 cases and 314,193 controls for LUAD, and 1,510 cases and 314,193 controls for LUSC. We obtained pQTL data from 7 large-scale proteomic studies 16-22. These pQTLs were selected based on the following criteria: (i) single nucleotide polymorphisms (SNPs) associated with plasma proteins levels at a genome-wide significance level (*P* < 5 × 10^-8^); (ii) exclusion of SNPs within the Major Histocompatibility Complex (MHC) region (chr6:25.5-34.0Mb); (iii) SNPs that are independent of each other, with linkage disequilibrium (LD) clumped at r^2^ < 0.001 within 10,000 kb window using the 1000 Genomes reference panel; (iv) utilization of F-statistics to evaluate the potential influence of weak instrument bias on the estimated effects of causal associations, wherein R^2^ denotes the variance explained by the IVs 23, 24. To eliminate duplicate proteins, we selected those with the highest cumulative of R^2^ values.

In total, 13,236 SNPs representing 4,853 unique plasma proteins were included in the analysis. Details regarding the data sources utilized in this study are provided in Table S1-S2. The instrumental variables are listed in Table S3.

### Proteome-wide MR analyses

We treated plasma proteins as exposures and the two subtypes of NSCLC as outcomes. The "TwoSampleMR" package 25 was used to conduct MR analysis in R. For proteins with a single SNP instrument, the Wald ratio method was employed to estimate the causal effect of circulating protein levels on the development of lung cancer. For the primary MR analyses, we applied the random-effects inverse variance weighted (IVW) method, which is appropriate for estimating MR effects for proteins with multiple SNP instruments. The MR-Egger, weighted median, simple mode, and weighted mode methods were also employed as sensitivity analyses to complement the IVW method for univariable models 26. MR-Egger regression was utilized to assess horizontal pleiotropy in MR analysis. We also conducted Cochran's Q test to evaluate heterogeneity among SNPs and assess the consistency of MR assumptions and analyses. The *P* value was adjusted using the false discovery rate (FDR) procedure; an adjusted *P* value (adj. *P*) of < 0.05 was considered statistically significant. Additionally, a *P* value < 0.05 was defined as the nominal significance level. Replication MR analyses were also conducted for the identified plasma proteins using LUAD and LUSC GWAS data from FinnGen; a *P* value < 0.05 was considered statistically significant in the replication group.

Identified protein markers considered as causal inferences must satisfy the following criteria. (i) The MR results using the Wald ratio or IVW methods show a nominally significant association with LUAD or LUSC risk (*P* value < 0.05). (ii) The Wald ratio or IVW methods, or weighted median, exhibit a significant association with LUAD or LUSC risk after the Benjamini-Hochberg test (adj. *P* < 0.05). (iii) The MR-Egger regression test suggests the absence of horizontal pleiotropy. All these procedures were carried out using R software version 4.3.0.

### Bayesian colocalization analyses

To investigate whether the observed association signals in two traits (trait 1: identified proteins, trait 2: NSCLC-related traits) are consistent with a shared genetic variant, we employed Bayesian colocalization analyses using the "coloc" package 27. This analysis encompassed the posterior probabilities of five hypotheses: (i) no causal genetic variant exists for either trait in the genomic locus (H0); (ii) a single causal genetic variant for trait 1 (H1); (iii) a single causal genetic variant for trait 2 (H2); (iv) two different causal genetic variants for both traits (H3); (v) shared causal genetic variants for both traits (H4). A posterior probability exceeding 90% for H4 (PPH4) was considered strong evidence, suggesting that the proteins and NSCLC potentially shared identical genetic variants.

### Summary-data-based MR (SMR) analyses and reverse MR analyses

Additionally, the SMR test was utilized to confirm the causal relationship between proteins and NSCLC risk 28. The heterogeneity in dependent instruments (HEIDI) test was conducted to perform a sensitivity analysis using SNPs located within a defined genomic region. This analysis aimed to determine whether the association between specific proteins and NSCLC risk arises from shared genetic variants rather than genetic linkage. A *P* value < 6.25×10^-3^ (0.05/8) was set as the significance threshold for the SMR analysis. Furthermore, a *P* value > 0.05 in the HEIDI test indicated that the association between the protein and NSCLC was not influenced by linkage disequilibrium. The SMR and HEIDI tests were conducted using the SMR 1.3.1 software 28.

We investigated the reverse causality relationship between the potential influence of LUAD and LUSC on protein levels. Proteins identified in the primary analyses were examined utilizing five MR methods (IVW, MR-Egger, weighted median, simple mode, and weighted mode). Furthermore, we considered GWAS significant genetic variants (*P* < 5 × 10^-08^) and independent genetic variants (clumped at r^2^ < 0.001), with an adjusted *P* value < 0.05 established as the threshold for statistical significance.

### Single cell‑type expression analyses and protein-coding genes expression pattern analyses

We further investigated the gene expression levels of plasma proteins across different cell types, specifically targeting those with a causal role in lung cancer development. This analysis leveraged single-cell RNA sequencing (scRNA-seq) data from the two major histopathological subtypes of NSCLC, and the adjacent non-cancerous tissue. The scRNA-seq data were sourced from the Gene Expression Omnibus (GEO) database. GSE149655 contains two LUAD tissue samples and two distal normal tissue samples. Additionally, we acquired two LUSC tissue samples, GSM3635278 and GSM3635285, from GSE127465. Using the “Seurat” package 29 in R, we implemented a standard workflow for data preprocessing and cell clustering based on the raw scRNA-seq data. The datasets for the two NSCLC subtypes and normal lung tissue underwent individualized analysis. During this process, genes with expression counts fewer than three in a single cell and cells with fewer than 200 unique features were excluded. Subsequently, we employed the NormalizeData and ScaleData functions to normalize and scale the transcripts per million (TPM) of RNA. The optimal number of principal components (PCs) for further analysis was determined using the RunPCA function, supplemented by constructing Elbow plots for each dataset. Cell clustering was conducted using the FindNeighbors and FindClusters functions, and non-linear dimensional reduction was performed using the RunUMAP function. To annotate various cell types, the “SingleR” package 30 was utilized with reference datasets HumanPrimaryCellAtlasData and BlueprintEncodeData. Utilizing GEPIA, a platform that combines extensive data from TCGA cancer and GTEx normal tissues, we created box plots to visualize the distribution of protein-coding genes in normal lung tissue and in LUAD or LUSC cancer tissues 31.

### GeneMANIA and Metascape analyses and proteins druggability evaluation

The GeneMANIA platform 32 (https://genemania.org/) was employed to investigate the potential genetic interactions and functions of the identified protein-coding genes. We also employed the Metascape database (http://metascape.org/) to conduct gene ontology (GO) biological process enrichment analysis. Drug repositioning, which involves utilizing the molecular structure, therapeutic indications, and adverse effects of an existing drug, represents a promising strategy for developing novel therapeutic functions. This approach has the potential to significantly reduce the costs and time associated with the discovery and approval of new treatments for various diseases 33. To determine whether the identified plasma proteins could serve as viable therapeutic targets for NSCLC, we searched three widely used drug-related databases: ChEMBL, DrugBank, and the Therapeutic Target Database (TTD). We documented the specific drug-gene interactions and outlined the drug development processes in this study.

### Protein associations with other traits

To deepen our understanding of the specificity of the identified cancer risk proteins, we undertook additional analytical steps using PheWAS and consulted several public databases. Our goal was to gather comprehensive insights into the potential risks or benefits associated with modulating the expression of theses protein in human populations. Firstly, we evaluated the score of the probability of being loss-of-function intolerant (pLI) for each cancer risk protein on the Exome Aggregation Consortium platform using the gnomAD browser. Secondly, we explored the UK Biobank using Genebass, which included exome-sequencing studies, rare-variant association studies, and Mendelian genetics research. To conduct a thorough assessment of the horizontal pleiotropy of potential drug targets and their possible side effects, we conducted a PheWAS using the AstraZeneca PheWAS Portal (https://azphewas.com/) 34. The study utilized a dataset from the UK Biobank, comprising approximately 15,500 binary and 1,500 continuous phenotypes derived from a subset of nearly 450,000 exome sequencing participants. To ensure the robustness of our findings, we applied rigorous statistical corrections and set a stringent threshold of 2E-9 (the default in the AstraZeneca PheWAS Portal) to mitigate the likelihood of false positives.

## Results

### Causal effects of 8 plasma proteins on NSCLC risk

**Figure 2:**
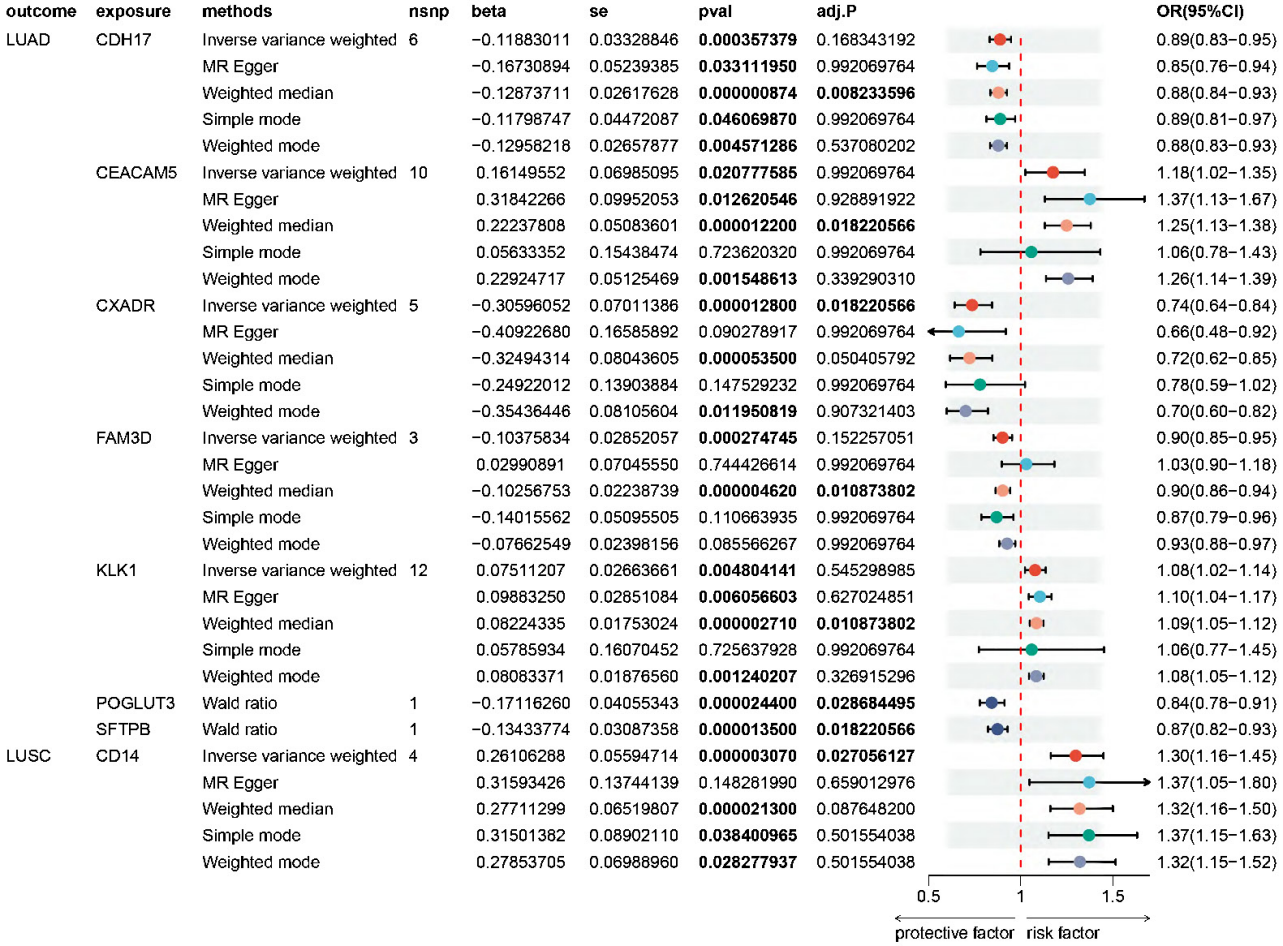
** Forest plot of Mendelian randomization (MR) analyses on the association of plasma proteins and the risk of non-small cell lung cancer.** MR point estimates and 95% confidence intervals (CIs) are provided for the 8 specific causal proteins in association with the risk of LUAD and LUSC. An adjusted *P* value (adj. *P*) below 0.05 indicated statistical significance, while a *P* value < 0.05 was set as the threshold for nominal significance level. OR, odds ratio; CIs, confidence intervals; LUAD, lung adenocarcinoma; LUSC, lung squamous cell carcinoma. Full name of proteins: CDH17, Cadherin-17; CEACAM5, Carcinoembryonic antigen-related cell adhesion molecule 5; CXADR, Coxsackievirus and adenovirus receptor; FAM3D, Family with sequence similarity 3, member D; KLK1, Kallikrein-1; POGLUT3, Protein O-glucosyltransferase 3; SFTPB, Pulmonary surfactant-associated protein B; CD14, Monocyte differentiation antigen CD14.

Proteome-wide MR analysis genetically identified eight plasma proteins associated with NSCLC, with seven linked to LUAD and one to LUSC. These proteins comprise Cadherin-17 (CDH17), Carcinoembryonic antigen-related cell adhesion molecule 5 (CEACAM5), Coxsackievirus and adenovirus receptor (CXADR), Family with sequence similarity 3, member D (FAM3D), Kallikrein-1 (KLK1), Protein O-glucosyltransferase 3 (POGLUT3), Pulmonary surfactant-associated protein B (SFTPB), and CD14. Higher levels of CDH17 (odds ratio (OR) = 0.89; 95% confidence interval (CI) 0.83-0.95), CXADR (OR = 0.74; 95% CI 0.64-0.84), FAM3D (OR = 0.90; 95% CI 0.85-0.95), POGLUT3 (OR = 0.84; 95% CI 0.78-0.91), and SFTPB (OR = 0.87; 95% CI 0.82-0.93) were associated with a reduced risk of LUAD. Conversely, CEACAM5 (OR = 1.18; 95% CI 1.02-1.35) and KLK1 (OR = 1.08; 95% CI 1.02-1.14) were linked to an elevated risk of LUAD. Furthermore, higher levels of CD14 (OR = 1.30; 95% CI 1.16-1.45) were associated with an increased risk of LUSC (Figure 2, Table S4-S6).

We validated our primary findings in the replication stage. Five proteins were successfully confirmed in the FinnGen cohort. The OR (95% CI) of NSCLC per SD increase in genetically predicted levels of protein was 0.73 (0.54-0.99) for CXADR, 0.85 (0.74-0.97) for FAM3D, 1.08 (1.00-1.17) for KLK1, 0.65 (0.51-0.83) for POGLUT3 in LUAD, and 1.53 (1.23-1.91) for CD14 in LUSC (Figure S1-S2, Table S7-S8).

### Sensitivity analyses for non-small cell lung cancer causal proteins

To further scrutinize the robustness of our primary MR analyses, Cochran's Q test was performed to assess heterogeneity across the identified eight causal proteins using the IVW or MR-Egger method. A significance level of *P* ≤ 0.05 would indicate the presence of heterogeneity, leading to the adoption of a random-effects IVW approach. Notably, four out of the eight proteins (CDH17, CXADR, FAM3D, CD14) exhibited no evidence of heterogeneity. Moreover, no signs of horizontal pleiotropy were identified in any of the eight proteins, as determined by the MR-Egger intercept method (*P*_pleiotropy_ > 0.05) (Table S4). During the replication stage, no heterogeneity or pleiotropy of the eight proteins was observed in either LUAD or LUSC (*P*_ heterogeneity_ > 0.05, *P*_pleiotropy_ > 0.05) (Table S7, S8).

For investigating potential reverse causality between the identified plasma proteins and different pathological types of NSCLC, we identified LUAD and LUSC as exposures and eight proteins as outcomes. Bidirectional MR analyses provided no evidence of a reverse causal relationship between the identified plasma proteins and different pathological types of NSCLC (Table S10).

### Causality Verification through Colocalization and SMR Analyses

Among the eight causal proteins identified through proteome-wide MR, POGLUT3 and SFTPB stand out, supported by robust evidence of genetic colocalization, with a PPH4 > 90% (Table 1, Table S9). This suggests a high likelihood of a shared causal variant influencing both the protein level and the risk of LUAD.

To distinguish pleiotropy from linkage and validate the primary proteome-wide MR findings, we conducted the SMR and HEIDI tests utilizing proteins with complete summary-level data. Table 1 indicates that only the protein SFTPB successfully passed both the SMR test (*P* < 6.25×10^-3^) and the HEIDI test (*P* > 0.05). This result suggests the lack of pleiotropy in the SNPs associated with SFTPB.

### Cell‑type specificity expression in the NSCLC tissue

**Figure 3:**
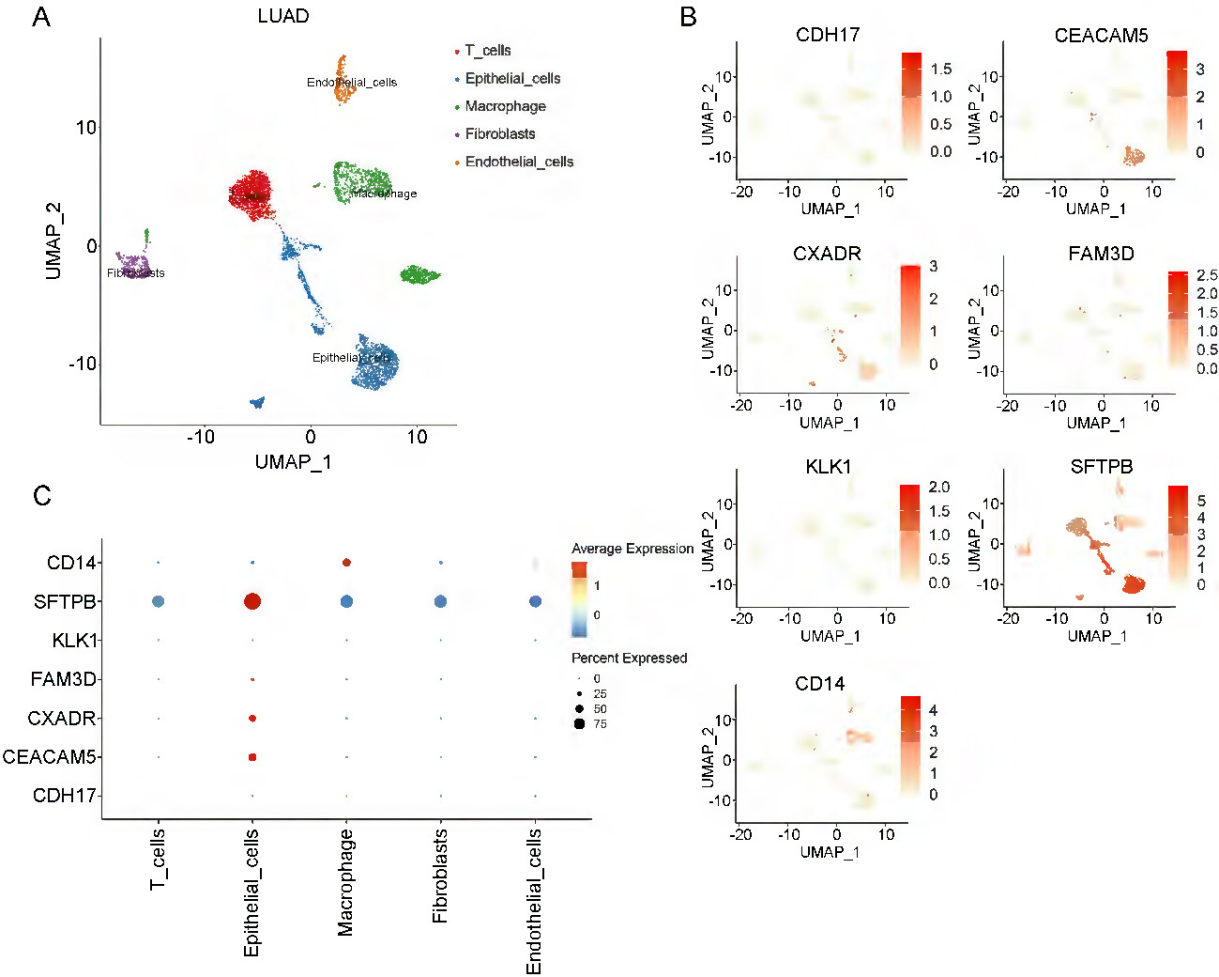
** Single-cell data analyses of lung adenocarcinoma.** A: The LUAD cell clusters were categorized into five distinct cell types; B and C display the expression patterns of the identified causal proteins within each cell type.

**Figure 4:**
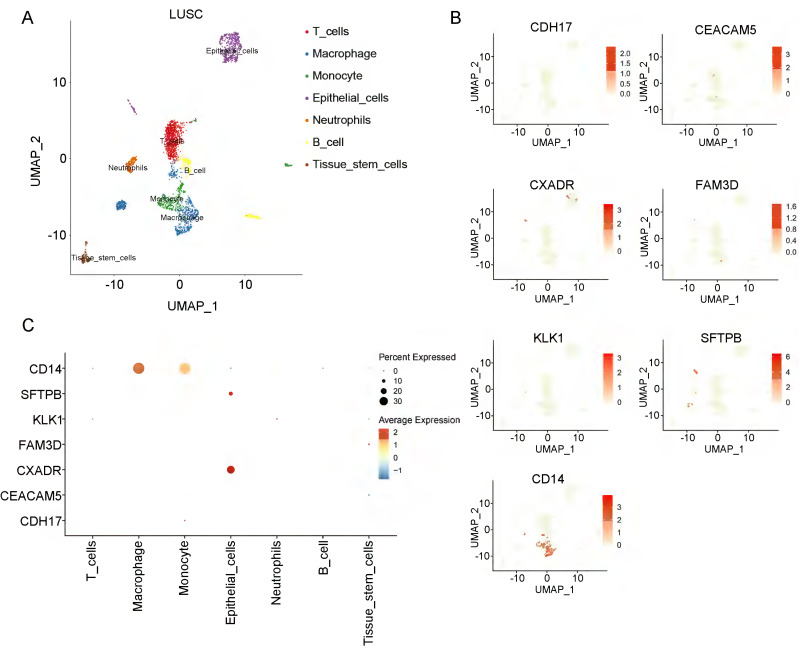
** Single-cell data analyses of lung squamous cell carcinoma.** A: The LUSC cell clusters were categorized into seven distinct cell types; B and C illustrate the expression of the identified causal proteins in each cell type.

To investigate the cell-type specificity of coding genes corresponding to the 8 plasma proteins in NSCLC tissue, we performed single-cell data analyses using the GEO database. In LUAD tumor tissue, using the FindCluster() function, we identified 13 clusters and annotated 5 cell types (T cells, epithelial cells, macrophages, fibroblasts, and endothelial cells) (Figure S3A, Figure 3A). Except for the *POGLUT3* gene, we detected 7 of the 8 protein-coding genes in LUAD tissue. Figure 3 (B-C) shows the single-cell expression of these 7 coding genes in each cluster. *CEACAM5*, *CXADR*, *FAM3D*, and *SFTPB* were mainly enriched in epithelial cells, and *CD14* was associated with macrophages, whereas *CDH17* was deficient in all clusters. We annotated 7 cell types (T cells, macrophages, monocytes, epithelial cells, neutrophils, B cells, and tissue stem cells) in LUSC tissue (Figure 4A, Figure S3B). *CXADR* and *SFTPB* were mainly enriched in epithelial cells, while *CD14* was enriched in both macrophages and monocytes (Figure 4B-4C). Moreover, in normal lung tissue, 6 protein-coding genes had expression data, while *POGLUT3* and *CDH17* were undetected. *CXADR* and *SFTPB* were mainly enriched in epithelial cells, while *FAM3D* and *CD14* were mainly enriched in endothelial cells (Figure S4). The expression levels of these 6 protein-coding genes in both NSCLC and normal tissue were also depicted in Figure S5.

### GeneMANIA analyses and druggability evaluation for candidate protein targets

**Figure 5:**
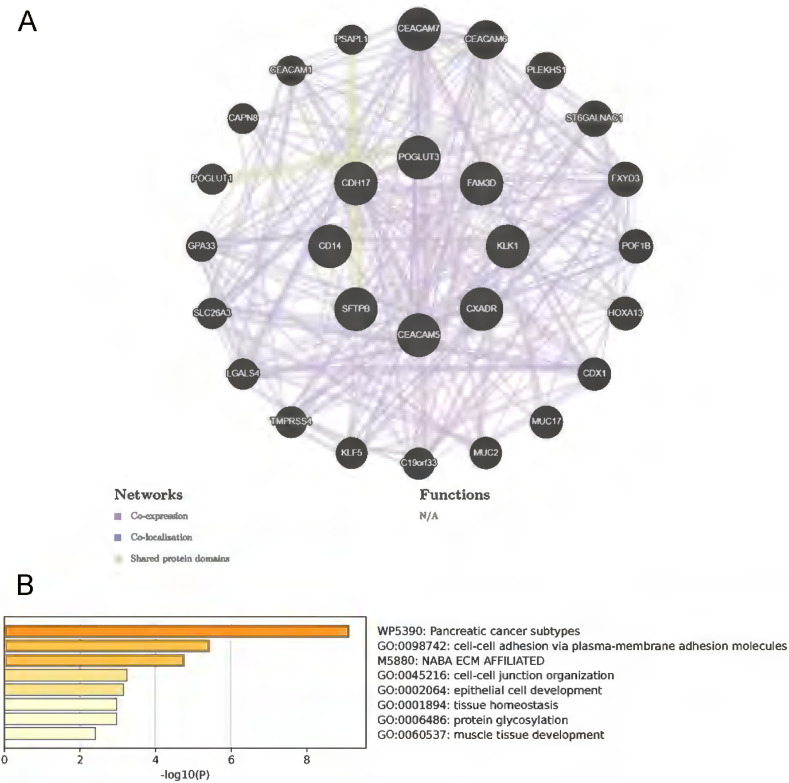
** The GeneMANIA and Metascape analyses for the eight protein-coding genes.** A: Eight identified protein-coding genes were placed in the inner circle, while twenty genes associated with them were situated in the outer circle. B: Metascape provides a bar graph for visualizing Gene Ontology (GO) enrichment analysis and interpreting metabolomic data related to the identified protein-coding genes.

We employed GeneMANIA to construct a comprehensive gene network by inputting the 8 protein-coding genes of interest. This analysis identified 20 correlated genes, predicated based on co-expression, co-localization, and shared protein domains. This expanded network, as depicted in Figure 5A, encompasses about a total of 510 interaction links. These interactions are categorized into co-expression (90.73%), co-localization (5.44%) and common protein structural domains (3.83%). A functional analysis of the network elucidates the roles of the drug targets and their associated genes, revealing significant involvement in cell-cell adhesion, tissue homeostasis, and protein glycosylation processes (Figure 5B).

In the assessment of the druggability and drug development potential of the eight candidate plasma proteins, we identified three proteins (CEACAM5, KLK1, CD14) that have been acknowledged as druggable targets (Table S11). CEACAM5, has been the focus of clinical trials for tusamitamab ravtansine, a drug designed for a spectrum of cancer types. Promising data from non-squamous NSCLC patients indicated a correlation between the antitumor activity and the expression level of CEACAM5 (Clinical trial information: NCT02187848; Registration Date: 2014-07-11) 35. Drugs targeting KLK1 have been researched for various applications, including lanoteplase for myocardial infarction, aprotinin for reducing bleeding and transfusion needs during surgery, nafamostat for anticoagulant therapy in acute kidney injury and liver transplantation scenarios, and aniline for the treatment of multiple myeloma. Drugs targeting CD14 have been applied in the treatment of sepsis with atibuclimab and autoimmune diseases, such as psoriasis and ulcerative colitis with the example of VB-201.

### Identifying loss-of-function variants and PheWAS analyses for causal proteins in non-small cell lung cancer

In our study, seven of the cancer risk proteins exhibited pLI scores below 0.1, implying a high tolerance for loss of function (LOF) variation. In contrast, CXADR showed a slightly lower intolerance of LOF variation, with a pLI score of 0.34. We conducted a gene-level PheWAS analysis using an extensive dataset from the AstraZeneca PheWAS Portal 34, which included 17,361 dichotomous and 1,419 quantitative phenotypes. As detailed in the Table S12, none of the eight drug targets showed significant associations with other traits or diseases at the gene level, with a rigorous significance threshold of *P* < 1E-05 for genomic association. We observed only limited evidence for the association of pLOF variants in cognate genes for cancer risk proteins with other traits, none of which were cancer endpoints. The pLOF burden in CXADR was associated with a lack of interest in doing things (*P* = 3.39^-5^, Beta = 2.82^-2^) and back pain (*P* = 8.88^-5^, Beta = 1.7^-1^), and FAM3D pLOF burden with chickenpox (*P* = 6.05^-5^, Beta = 1.69^-1^). Additionally, protein-damaging missense variation in CXADR was linked to cervical spondylosis (*P* = 5.19^-5^, Beta = 3.88^-2^), FAM3D was associated with B44 aspergillosis (*P* = 3.35^-5^, Beta = 7.14^-2^), and POGLUT3 with xenograft replacement of aortic valve (*P* = 3.18^-5^, Beta = 4.31^-2^), stroke (*P* = 3.22^-5^, Beta = 1.45^-2^) and vascular/heart problem (*P* = 6.65^-5^, Beta = 1.31^-2^). This finding suggests a low probability of substantial side effects associated with drugs targeting these genes or the presence of significant horizontal pleiotropy.

## Discussion

In this study, we systematically evaluated the causal relationship between plasma proteins and NSCLC. Using pQTL data from up to 4,853 plasma proteins, we identified eight proteins that likely play a role in the etiology of NSCLC. Specifically, genetically determined lower levels of proteins such as CDH17, CXADR, FAM3D, POGLUT3, and SFTPB, along with higher levels of CEACAM5 and KLK1, were associated with increased risk of LUAD, while higher levels of CD14 were associated with increased risk of LUSC. Additionally, five proteins (CXADR, FAM3D, KLK1, POGLUT3, CD14) were corroborated in external cohorts; two proteins (POGLUT3, SFTPB) were verified by Bayesian colocalization; and one protein (SFTPB) was identified through SMR and HEIDI tests, underlining their potential therapeutic relevance. Subsequently, bidirectional MR was conducted and no proteins revealed reverse causality. We further verified the differential expressions of these protein-coding genes in various cell types, including epithelial cells, macrophages, monocytes, and endothelial cells. We also identified causal proteins that mediate the therapeutic effects of specific drugs that may lead to drug repurposing. Moreover, we identified proteins as novel targets that were previously unexplored in the context of NSCLC therapy, thereby paving the way for new investigational pathways. Lastly, we employed phenome-wide association analysis to delve deeper into the potential pleiotropic effects of the target genes and to foresee possible drug side effects, ensuring a comprehensive evaluation of our findings.

The significance of our findings lies in their extension of current evidence associating these proteins with lung cancer, based on studies of gene polymorphisms, mRNA, or protein levels. It is notable that SFTPB was identified based on the strongest evidence from colocalization, SMR and HEIDI tests. SFTPB is an essential protein for maintaining normal lung function 36. SFTPB is first synthesized as a hydrophilic 42-kD protein by type 2 alveolar pneumocytes and nonciliated bronchiolar cells in the form of pro-SFTPB. Following its synthesis, pro-SFTPB rapidly undergoes proteolytic cleavage by cysteine proteases within the endoplasmic reticulum, resulting in the production and secretion of a 9-kD non-collagenous hydrophobic SFTPB, which represents the functional mature form of the protein. Research has indicated that pro-SFTPB may serve as a potential biomarker for lung cancer, with elevated levels possibly signifying an increased risk of lung cancer 37, 38. The mature SFTPB has also been recognized as a potential biomarker for identifying metastatic lymph node involvement in patients with NSCLC 39. Existing assays are unable to differentiate between precursor and mature forms; however, numerous studies have established that total SFTPB is associated with lung cancer prognosis 37. Our study provides population-level evidence confirming the causal relationship between reduced SFTPB protein levels and NSCLC risk, suggesting that SFTPB agonists could be a promising avenue for therapeutic prevention in high-risk individuals with lung cancer.

KLK1, a member of the kallikrein-related peptidase family, has been implicated in regulating vascular tone, inflammation, and tumor microenvironment 40, 41. Our findings align with recent studies suggesting a causal role for KLK1 in cancer prognosis and carcinogenesis, indicating that drugs targeting KLK1 may offer an avenue for lung cancer prevention. CEACAM5, a cell-surface glycoprotein, has emerged as a promising therapeutic target in preclinical models 42. Our findings support the idea that CEACAM5 may be a risk factor for LUAD, suggesting that high-risk individuals with LUAD may benefit from targeted therapeutic interventions. CD14, a co-receptor involved in innate immune responses, has been linked to disease aggressiveness in different cancers 43-45. Elevated levels of CD14 have been observed in NSCLC patients 46. Our research supports the role of CD14 in promoting LUSC carcinogenesis, however, the mechanisms are yet to be elucidated. POGLUT3, an O-glucosyltransferase, plays a pivotal role in targeting secreted proteins crucial for the assembly and function of the extracellular matrix 47, 48. Our findings indicate that a genetic predisposition to elevated POGLUT3 levels is linked to reduced LUAD risk, underscoring the necessity for additional epidemiological studies and experimental investigations. While the effect sizes observed in our MR analysis—specifically, the association between a 1- SD increase in CEACAM5 levels and LUAD risk (OR = 1.18, 95% CI 1.02-1.35)—are indeed more modest than those of established epidemiological risk factors, such as smoking (e.g., a 15-fold increased risk for childhood-onset smokers in the UK Biobank 49), this observation aligns with the biological role of plasma proteins as intermediate mediators in complex disease pathways. Furthermore, the synergistic potential of multiple proteins (e.g., CDH17 and CEACAM5) may amplify risk through either additive or interactive mechanisms, which is consistent with the rationale for multi-target combination therapies in cancer 50. The lack of statistical significance for CEACAM5, CDH17, and KLK1 in the FinnGen cohort may stem from population differences or environmental interactions. The distinct genetic ancestry of the FinnGen cohort (Finnish-specific haplotype frequencies) 51 and its environmental context likely contribute to challenges in replication.

Proteins serve as essential targets for drug development. We identified three proteins that are the focus of drugs currently under investigation in phase I clinical trials or higher, indicating that their potential for drug-ability is actively being evaluated. Notably, the majority of drugs targeting cancer risk proteins are typically either monoclonal antibodies or small molecular inhibitors (SMIs). For example, labetuzumab, a monoclonal antibody, directly inhibits CEACAM5 and is utilized in the treatment of colorectal cancer 52. KLK1 is inhibited by several SMIs in the treatment of cardiovascular diseases 53, while the anti-CD14 antibody atibuclimab is administered to patients with amyotrophic lateral sclerosis 54. The identification of CEACAM5, KLK1, and CD14 as established drug targets for NSCLC presents significant therapeutic potential. These proteins are currently the focus of clinical trials and are involved in ongoing drug development efforts targeting both cancer and inflammatory diseases. Our study's findings further strengthen the argument for their role in NSCLC and suggest that these targets could be utilized in the development of novel treatment strategies, either individually or in combination with current therapeutic modalities, including immunotherapy, chemotherapy, or anti-angiogenic therapies. We propose that further exploration of these targets in clinical trials will facilitate the translation of these findings into effective therapeutic options for patients with NSCLC.

Our research demonstrates several strengths, such as a robust repository of plasma proteins, a substantial cohort of LUAD and LUSC cases, a minimal risk of reverse causation, and comprehensive colocalization analyses. These elements highlight the effectiveness of our methodology in elucidating the fundamental mechanisms governing NSCLC pathogenesis. The supplementary insights obtained from single-cell expression analysis, PPI studies, and drug target assessments have shed light on the potential pathogenic roles of candidate proteins in NSCLC. This has not only helped to delineate priority targets for drug development but also underscored the potential of our investigation to augment genetic screening methodologies for the early detection of NSCLC. Our work provides essential insights into the development of screening and preventative strategies, particularly for those with identified genetic predispositions. Furthermore, we underscore the potential for drug repurposing, acknowledging that high-risk proteins not currently the subject of any drug intervention may offer promising avenues for future drug discovery. Additionally, the identification of previously uninvestigated cancer-associated proteins unveils novel opportunities for transformative approaches in cancer treatment and prevention.

Our study presents several limitations that necessitate acknowledgment. Firstly, our study lacked a comprehensive assessment of the entire proteome, as it was restricted to measuring proteins exclusively via blood-based multiplex affinity platforms. Secondly, our analysis concentrated solely on individuals of European descent, thereby hindering the generalizability of our findings in the genetic landscape of diverse populations. To this end, we intend to utilize GWAS from East Asian populations, such as the China Kadoorie Biobank and Biobank Japan, to conduct cross-ethnic MR analysis in our future research. Thirdly, the statistical power might be insufficient in analyzing large cell lung cancer due to the absence of genome-wide association study (GWAS) data for this histological subtype of NSCLC. To evaluate the histological heterogeneity of NSCLC in greater detail, we intend to integrate single-cell multi-omics approaches in our subsequent studies. Fourthly, the GWAS summary data for LUAD and LUSC from public datasets exhibited imbalanced stratification across demographic covariates such as gender, age, and other crucial demographic factors. This imbalance constrained our ability to investigate potential causal links within distinct subgroups. Fifth, to address the absence of experimental validation, we plan to incorporate experimental validation through CRISPR-based protein knockdowns of SFTPB and CEACAM5 in NSCLC cell lines in our future work. Lastly, we conducted rigorous sensitivity analyses, including MR-Egger regression (*P*_pleiotropy_ > 0.05 for all proteins; see Table S4), weighted median methods, and Cochran's Q tests, to minimize bias from horizontal pleiotropy. However, due to the prevalent occurrence of horizontal pleiotropy in MR analysis, we were unable to sufficiently attenuate its impact in our study; nevertheless, this phenomenon may reveal alternative pathways associated with the traits under investigation.

## Conclusions

Our study identified several plasma proteins associated with NSCLC risk, providing novel insights into its etiology and highlighting potential targets for diagnostic and therapeutic advancements. Further experimental and clinical investigations are imperative to assess the efficacy and significance of the eight identified proteins, with the goal of developing preventive strategies and clinical approaches to address the anticipated increase in cancer prevalence.

## Supplementary Material

Supplementary figures and tables.

## Figures and Tables

**Table 1:** Summary results from Mendelian randomization (MR), colocalization, and SMR for 8 proteome-wide MR-identified proteins**.**

Protein	Protein full name	MR		Colocalization	SMR		
		Beta	P	PPH4>0.9	Beta	P	PHEIDI
CDH17	Cadherin-17	-0.12	3.57E-04	No	-	-	-
CEACAM5	Carcinoembryonic antigen-related cell adhesion molecule 5	0.16	2.08E-02	No	-	-	-
CXADR	Coxsackievirus and adenovirus receptor	-0.31	1.28E-05	No	-	-	-
FAM3D	Family with sequence similarity 3, member D	-0.10	2.75E-04	No	-0.129963	5.09E-02	0.4
KLK1	Kallikrein-1	0.08	4.80E-03	No	-	-	-
POGLUT3	Protein O-glucosyltransferase 3	-0.17	2.44E-05	Yes	-	-	-
SFTPB	Pulmonary surfactant-associated protein B	-0.13	1.35E-05	Yes	-0.135282	1.50E-05	0.3
CD14	Monocyte differentiation antigen CD14	0.26	3.07E-06	No	-	-	-

Abbreviations: HEIDI, heterogeneity in dependent instruments; MR, Mendelian randomization; PPH4, posterior probability exceeding 90% for H4; SMR, summary-data-based Mendelian randomization.

## Abbreviations

CDH17: Cadherin-17
CEACAM5: Carcinoem-bryonic antigen-related cell adhesion molecule 5
CXADR: Coxsackievirus and adenovirus receptor
FAM3D: Family with sequence similarity 3, member D
FDR: False discovery rate
GO: Gene ontology
GWAS: Genome-wide association study
HEIDI: Heterogeneity in dependent instruments
IVs: Instrumental variables
IVW: Inverse variance weighted
KLK1: Kallikrein-1
LD: Linkage disequilibrium
LOF: Loss of function
LUAD: Lung adenocarcinoma
LUSC: Lung squamous cell carcinoma
MHC: Major histocompatibility complex
MR: Mendelian randomization
NSCLC: Non-small cell lung cancer
PheWAS: Phenome-wide association analyses
pLI: Probability of being loss-of-function intolerant
POGLUT3: Protein O-glucosyltransferase 3
pQTL: Protein quantitative trait loci
RCTs: Randomized controlled trials
SFTPB: Surfactant-associated protein B
SMR: Summary-data-based Mendelian randomization
SNPs: Single nucleotide polymorphisms
