# Supplementary material for: Plasma Proteome Profiling Identifies Biomarkers and Potential Drug Targets for Non-Small Cell Lung Cancer
Source: Int J Med Sci. 2025 Sep 12;22(15):4036–48. doi: 10.7150/ijms.107109 (PMC12492361; doi:10.7150/ijms.107109)
Supplement: Supplementary file 1 — Supplementary figures and tables. [file ijmsv22p4036s1.zip › Supplementary materials/Supplementary Figure Legend.docx]

Figure S1. Forest plot of Mendelian randomization (MR) analyses on the association of plasma proteins and the risk of lung adenocarcinoma in the FinnGen cohort. A *P* value < 0.05 was considered statistically significant in the replication group.

OR, odds ratio; CIs, confidence intervals; LUAD, lung adenocarcinoma; LUSC, lung squamous cell carcinoma. Full name of proteins: CDH17, Cadherin-17; CEACAM5, Carcinoembryonic antigen-related cell adhesion molecule 5; CXADR, Coxsackievirus and adenovirus receptor; FAM3D, Family with sequence similarity 3, member D; KLK1, Kallikrein-1; POGLUT3, Protein O-glucosyltransferase 3; SFTPB, Pulmonary surfactant-associated protein B; CD14, Monocyte differentiation antigen CD14.

Figure S2. Forest plot of MR analyses on the association of plasma proteins and the risk of lung squamous cell carcinoma in the FinnGen cohort. A *P* value < 0.05 was considered statistically significant in the replication group.

Figure S3. Single cell data analyses of NSCLC.

A: Umap of the 13 cell clusters from lung adenocarcinoma; B: Umap of the 13 cell clusters from lung squamous cell carcinoma.

Figure S4. Single cell data analyses of normal lung tissue.

A: The cell clusters were divided into seven cell types in lung tissue; B and C show the expression of the identified causal proteins in each cell type.

Figure S5. The expression levels of identified protein-coding genes in NSCLC and normal lung tissue.

The levels of the 6 identified protein-coding genes and normal samples from GEPIA database. (**P* < 0.05).
