# Supplementary figures and images for: Plasma Proteome Profiling Identifies Biomarkers and Potential Drug Targets for Non-Small Cell Lung Cancer
Source: Int J Med Sci. 2025 Sep 12;22(15):4036–48. doi: 10.7150/ijms.107109 (PMC12492361; doi:10.7150/ijms.107109)

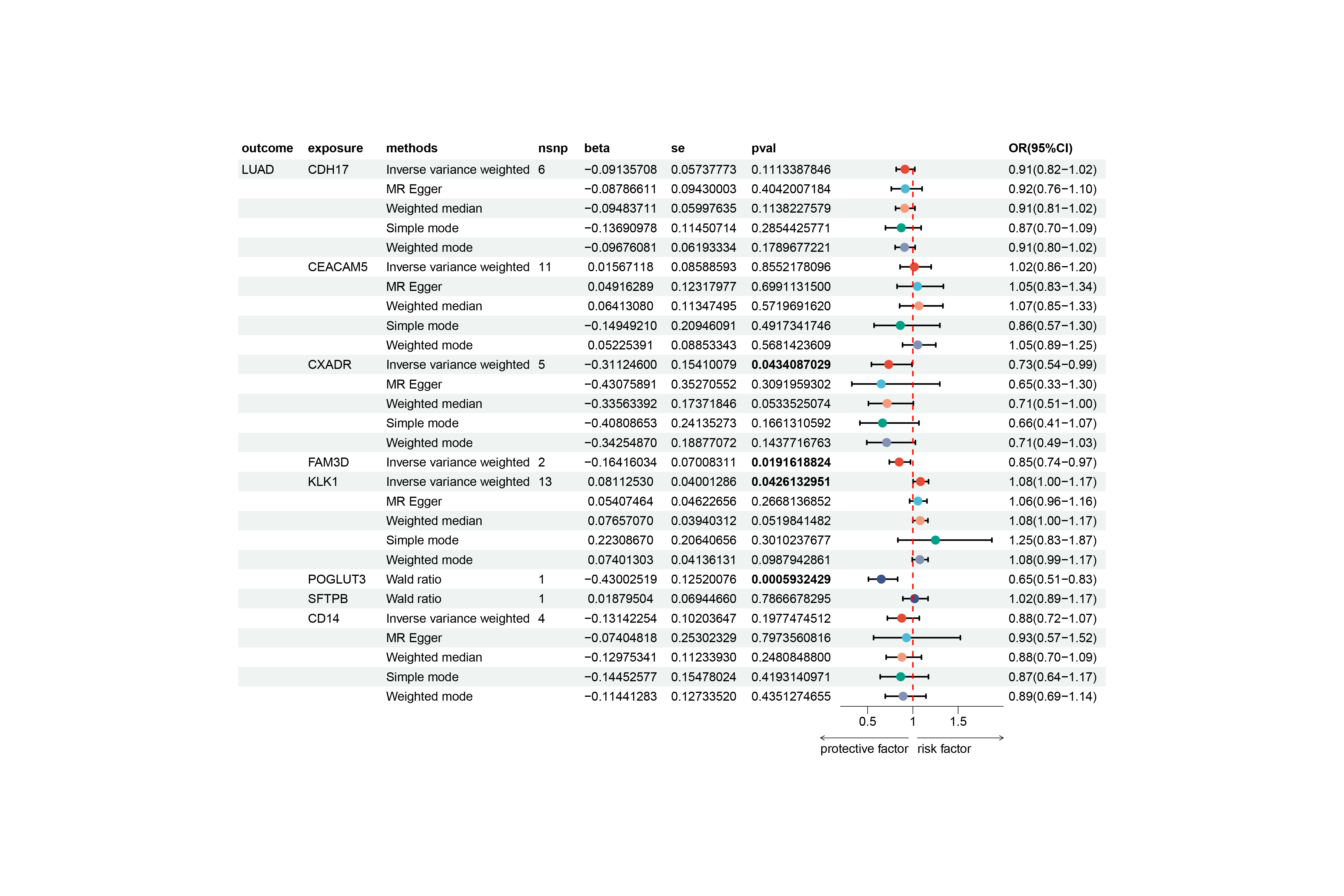

Supplement: Supplementary file 1 — Supplementary figures and tables. [file ijmsv22p4036s1.zip › Supplementary materials/Figure.S1.tif]

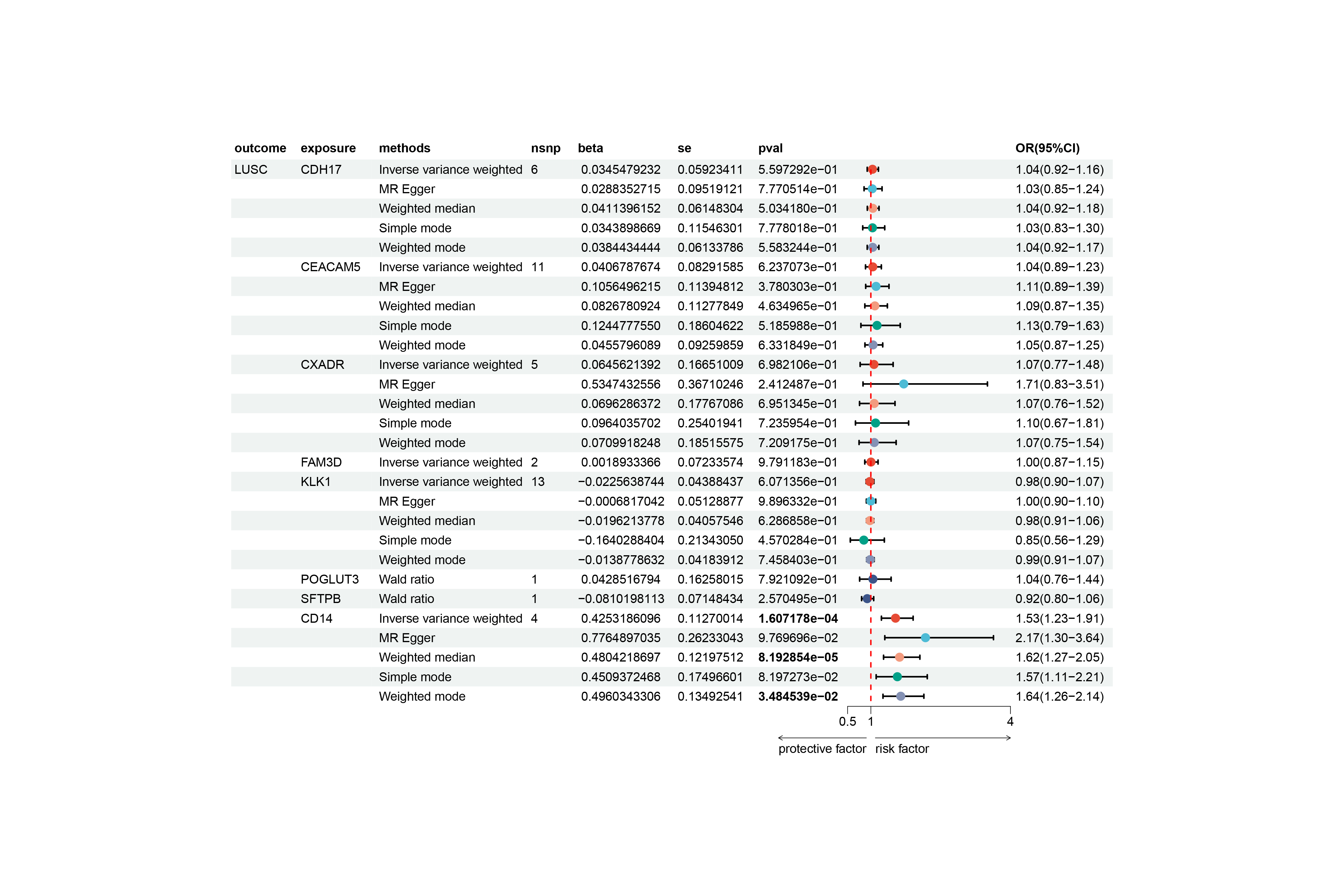

Supplement: Supplementary file 1 — Supplementary figures and tables. [file ijmsv22p4036s1.zip › Supplementary materials/Figure.S2.tif]

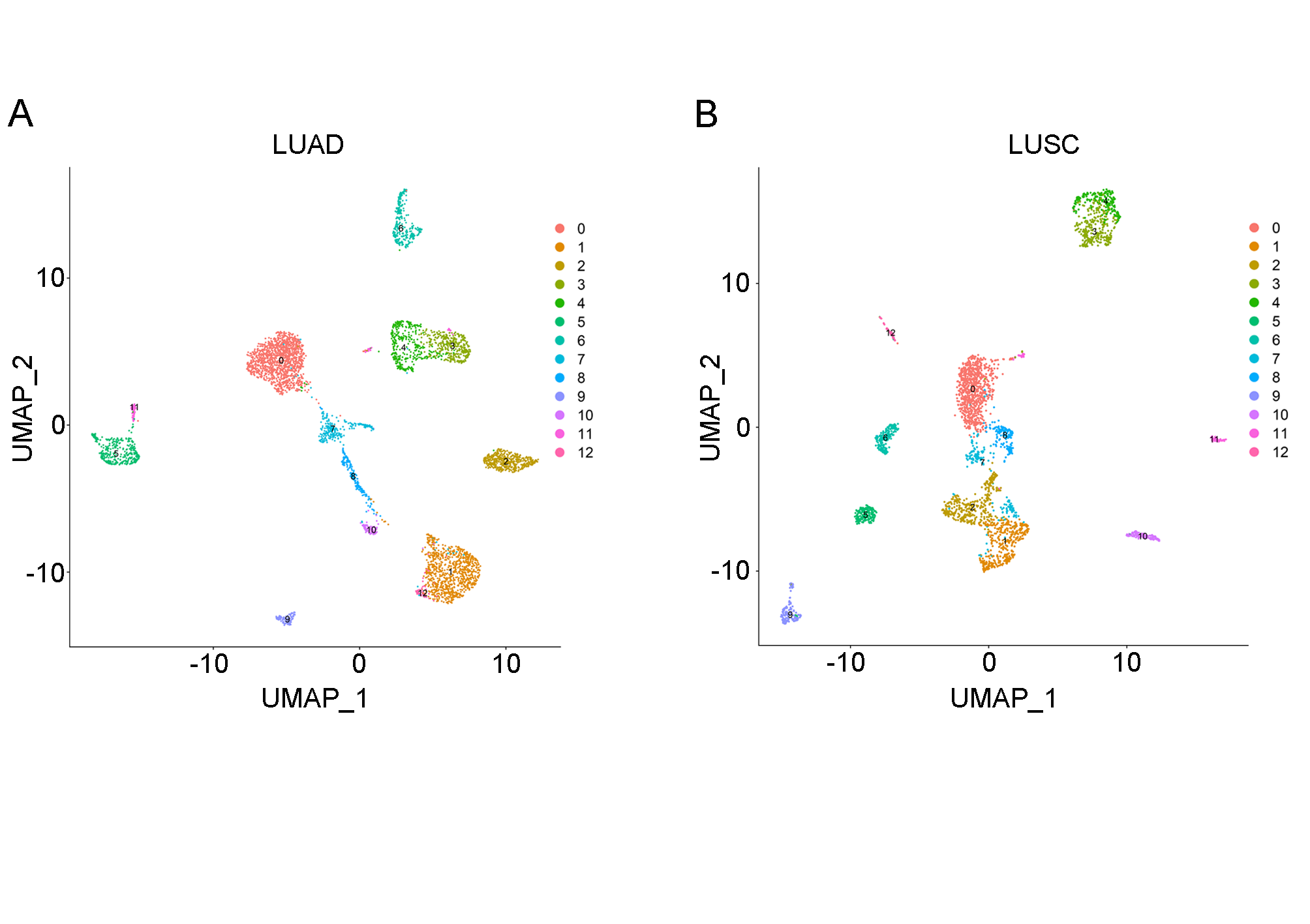

Supplement: Supplementary file 1 — Supplementary figures and tables. [file ijmsv22p4036s1.zip › Supplementary materials/Figure.S3.tif]

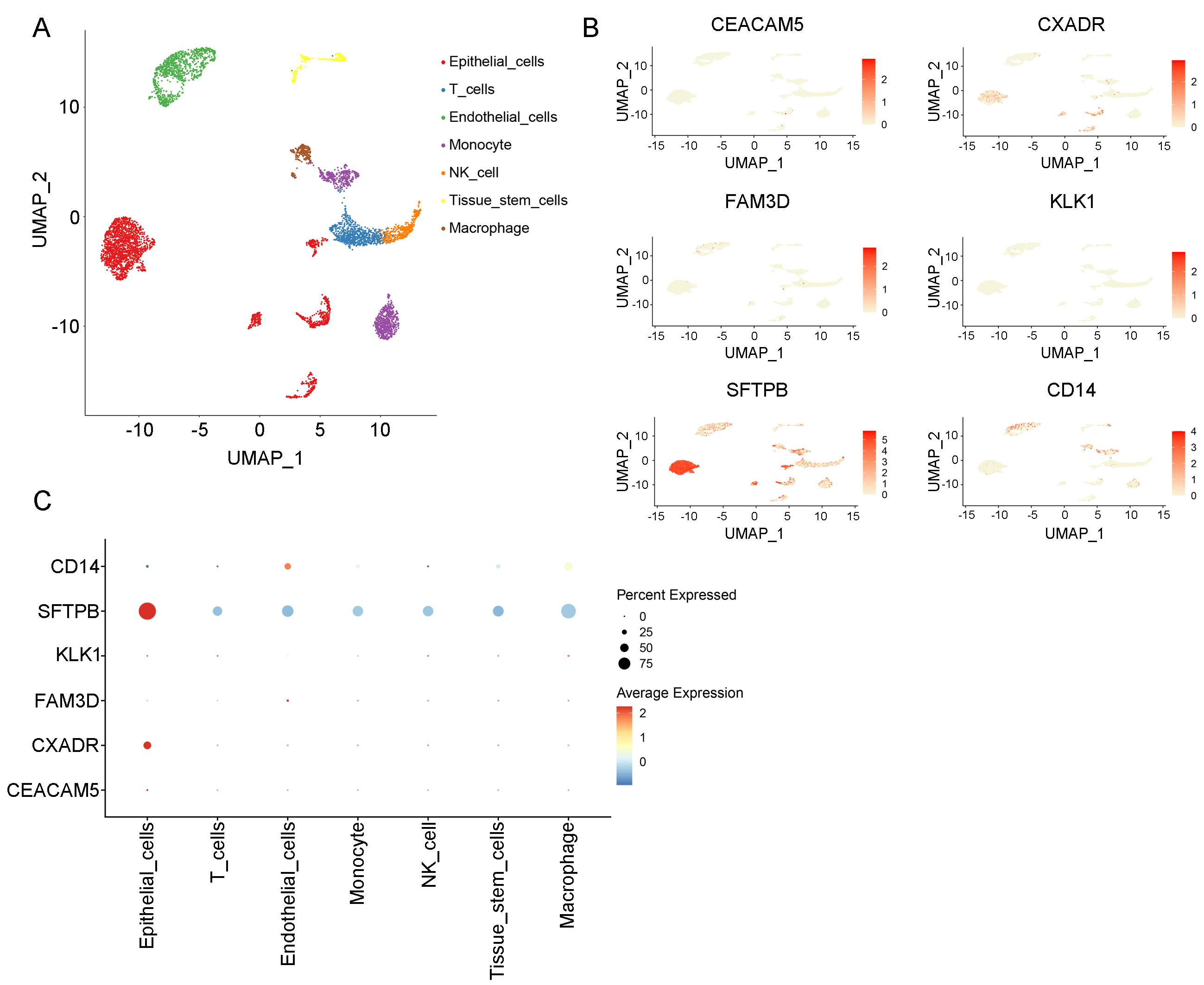

Supplement: Supplementary file 1 — Supplementary figures and tables. [file ijmsv22p4036s1.zip › Supplementary materials/Figure.S4.tif]

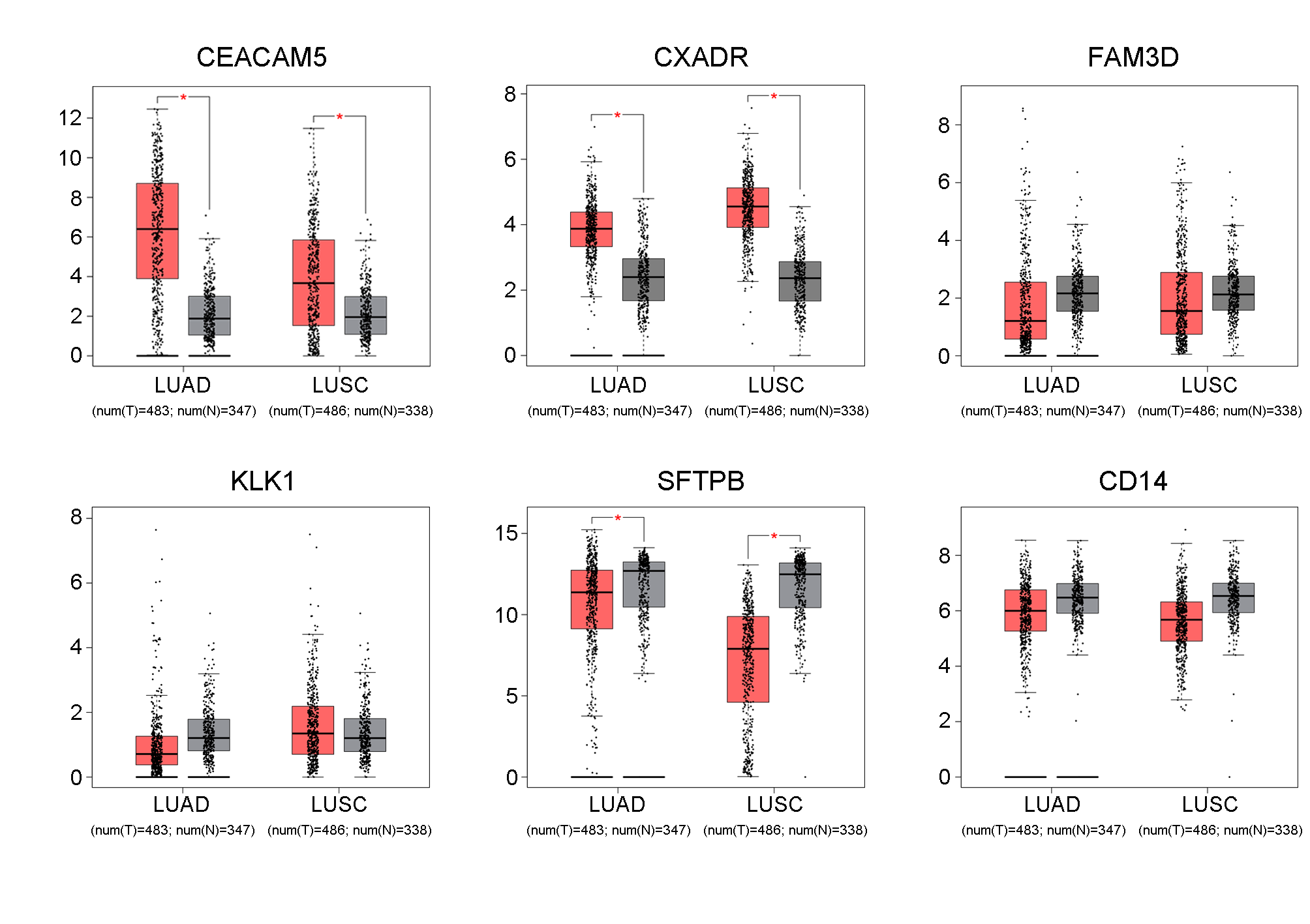

Supplement: Supplementary file 1 — Supplementary figures and tables. [file ijmsv22p4036s1.zip › Supplementary materials/Figure.S5.tif]
